# Research development of the relationship between thymidine phosphorylase expression and colorectal carcinoma

**DOI:** 10.7497/j.issn.2095-3941.2013.01.002

**Published:** 2013-03

**Authors:** Dian-Jun Ye, Ji-Min Zhang

**Affiliations:** Department of Gastrointestinal Surgery, The Second Affiliated Hospital of Guangzhou Medical University, Guangzhou 510260, China

**Keywords:** Thymidine phosphorylase, colorectal carcinoma, angiogenesis, 5’-deoxy-5-fluorouridine

## Abstract

Thymidine phosphorylase (TP) is a key enzyme that contributes to the composition and decomposition of pyrimidine nucleotides. TP seems homologous to platelet-derived endothelial cell growth factor, and its effects on inducing vascularization and anti-apoptosis are closely related to growth and metastasis of colorectal carcinoma. In addition, TP is a key enzyme that catalyzes the transformation from 5-fluorouracil (FU) prodrugs of 5′-deoxy-5-fluorouridine (5′-DFUR) to 5-FU. The activity of TP is closely related to the sensitivity of colorectal carcinoma cells to fluorouracil drugs and targeted therapy. Given the important functions of TP in growth, metastasis, tumor treatment, and prognosis, determining its expression mechanism is significant. This article summarizes the research development of TP expression in colorectal carcinoma, tumor neovascularization, cytotoxicity activation of 5′-DFUR, and colorectal carcinoma therapy.

## Introduction

Thymidine phosphorylase (TP) is a key enzyme that contributes in the metabolic process of pyrimidine nucleotides. TP has a structure similar to that of platelet-derived endothelial cell growth factor (PD-ECGF); therefore, these two constituents are considered to be the same substance. TP promotes angiogenesis and has important functions in growth, invasion, and metastasis of colorectal carcinoma^1^. TP also can selectively activate anticancer reagent 5′-deoxy-5-fluorouridine (5′-DFUR) into fluorouracil (5-FU), resulting in an inhibiting effect on cancer cells. Consequently, these drugs can have antitumor effects. Therefore, TP expression studies are important for investigating angiogenesis, invasion, and metastasis of colorectal carcinoma, as well as for chemotherapy.

## Biological property of TP

Friedkin discovered TP in a biological body in 1954. In 1975, Voytek and Blank separated and purified TP from *Escherichia coli* and *Salmonella*, which had a 45 kDa subunit dimer. In 1978, Kubilus was the first to purify a eukaryotic cell TP from the amniotic membrane chorion of a human. This eukaryotic cell TP was a 47 kDa-subunit dimer that had the same properties as *E. coli* TP. In 1987, Miyazono stimulated an aortic endothelial cell and obtained PD-ECGF in platelet-cleaving factors from the proliferation of endothelial cells. In 1992, Furukawa discovered that the 120 amino acid sequences in the cDNA of *E. coli* TP were identical to the sequences of PD-ECGF through mass cloning experiments. This discovery has drawn attention to the relationship between TP and PD-ECGF. In 1993, Sumizawa’s study showed that recombinant PD-ECGF possesses TP activity, and TP could promote angiogenesis activity. The PD-ECGF polypeptide could be recognized by anti-TP antibody. This result suggests that these two materials have a high degree of homology. In 1995, Miyadera purified human placenta TP and analyzed the amino acid sequences. He discovered that the N-terminal sequences of TP and PD-ECGF had slight differences, with some missed fragments in the course of purification. Therefore, TP and PD-ECGF were identified as transcription products from the same gene. To date, TP and PD-ECGF are considered similar substances that contribute in the metabolic process of pyrimidine nucleotides and promote angiogenesis inside the body.

TP is a homodimer that consists of two polypeptide chains. Its relative molecular mass and best-fit PH value are 55 kDa and 5.3, respectively. TP gene is located on the q13 region of chromosome 22. Its main biological functions are the following: (1) TP can reversibly catalyze thymidine dephosphorylation. This catalysis subsequently generates thymine and 2-deoxyribose-D-ribose-1-phosphate, which can maintain a stable level of thymidine inside the body, including thymidine degradation for carbon and energy source and nucleotide synthesis with thymine. Deoxyribose can be transferred from deoxynucleoside to other basic groups for new deoxynucleoside formulation^2^; (2) TP/PD-ECGF can stimulate chemotaxis and growth of endothelial cells in vitro and affects angiogenesis inside the body; (3) TP can catalyze the chemical reaction between drugs of anti-pyrimidine metabolism 5-fluorouracil (FU) and 1-phosphate-2-deoxyribose. The resulting 5-fluoro-2-deoxyuridine is involved in the metabolic process of 5-FU. The end product of metabolism, which has an anticancer activity, is then generated^3^; (4) TP is also the key metabolic enzyme of 5′-DFUR and CAP, which are the prodrugs of 5-FU. TP can promote 5′-DFUR and CAP and convert them into 5-FU to exert antitumor effects^3^.

## Relationship between TP and angiogenesis in colorectal cancer

Formation and development of malignant tumor generally rely on neovascularization. As an angiogenesis-promoting factor, TP has an important function in the growth, invasion, and metastasis of colorectal carcinoma. Inokuchi^4^ indicated that patients with high TP expression in colorectal carcinoma tissue have more new vessels in the cancer tissues and exhibit liver metastasis; bad prognosis could more easily occur in these patients. Using immunohistochemical staining method, Tsujianti^5^ studied the relationship between TP expression, vascular endothelial growth factor (VEGF), and microvessel density (MVD) in gastric cancer that the placenta percreta had infiltrated. The results showed that the tumors expressing positive TP and VEGF had the maximum MVD. By contrast, the tumors expressing negative TP and VEGF had the minimum MVD. Xia *et al.*^6^ studied the relationship between TP, VEGF expression, and clinicopathologic characteristics of 88 colorectal carcinoma specimens using immunohistochemical staining EnVision two-step method. Approximately 42.05% (37/88) and 23.86% (21/88) colorectal carcinoma tissues were TP positive and expressed VEGF, respectively. Almost no expression was observed in normal colonic mucosa. This result suggests that TP and VEGF may be necessary for the differentiation and development of cancer cells. Given the obviously lower expression of VEGF than TP, angiogenesis in colorectal carcinoma tissue possibly relies on TP to a greater degree than on VEGF. Therefore, angiogenesis mediated by TP is more likely the key factor of development and metastasis of colorectal carcinoma. TP is different from VEGF and other growth factors because no hydrophobic signal peptide exists at the N-terminal. This phenomenon is not a classical secretory protein. The enzymatic activity of TP can generate corresponding products to promote angiogenesis. Nishina *et al.*^3^ stated that TP can catalyze thymidine to obtain 2-deoxidization-D-ribose, and these metabolic processes can induce a mass of free radicals and create a local oxidized stress state. This state induces an expression of active factors, such as VEGF and IL-8. Angiogenesis in colorectal carcinoma is promoted in each functional pathway. This result suggests that TP expression promotes angiogenesis of colorectal carcinoma and contributes to growth, development, and metastasis of tumor. However, further studies are still needed to clearly elucidate the interactions between TP expression and other growth factors.

## TP expression in colorectal carcinoma

The expression of TP in colorectal carcinoma tissues is apparently higher than in the surrounding normal tissues. However, different findings exist on whether cancer cells or interstitial cells in carcinoma tissues express TP. Early studies have indicated that cancer cells could directly express TP in high level, as verified in gastric and breast cancers. In 2001, Kono used immunohistochemistry to compare colorectal cancer specimens by applying different anti-TP antibodies of 654-1 and 1 c6-203. Cancer cells were positively expressed in 20% and 60% of the 654-1 and 1C6-203 applied specimens, respectively, after immunohistochemical staining. The 654-1 antibody was mainly expressed in interstitial cells. In 2002, Tokunaga also studied the 1C6-203 antibody, and the results showed that TP was positively expressed in 68% of the colorectal carcinoma specimens. Yoshimoto *et al.*^7^ analyzed TP expressions in 61 colorectal carcinoma specimens. The result indicated that TP was mainly expressed in interstitial cells surrounding the tumor tissue or infiltrating the border of the tumor. Lymph node metastasis was also related to this phenomenon, which suggests that TP may be expressed by cancer cells, and then subsequently released to the infiltrating edge.

In recent years, studies have suggested that TP is mainly expressed by interstitial cells in the tumor tissue, especially the tumor-associated macrophage (TAM) on the tumor edge. In 1999, Ono concluded that when a tumor is present in an organism, cancer cells would release different kinds of chemokines, such as monocyte chemoattractant protein-1. These chemokines could induce migration of mononuclear cells toward the tumor location and differentiation of macrophages. The differentiation could generate mass of cytokines, such as VEGF, tumor necrosis factor (TNF), TNF-ɑ, interferon (INF), epidermal growth factor, and TP to induce angiogenesis. Zhang *et al.*^8^ performed TP enzyme assays and protein quantitative detections for 40 cases of colorectal carcinoma specimens; 6 colorectal carcinoma cell lines, namely, LS174T, Clone A, Colo320, CX-1, Lovo, and MIPIO1; and 2 macrophagic systems of THP-1 and U937. The methods used include enzyme-linked immunosorbent assay (ELISA) and immunohistochemical staining method. The results showed that the TP activity of colorectal carcinoma cell was evidently higher than that of the normal tissue. Minute and small amounts of TP proteins were detected in LS174T and Lovo, respectively; however, these proteins were not detected in the other four carcinoma cell lines. Up to 18.2 and 19.3 U/mg of TP proteins were detected in THP-1 and U937, respectively. This finding indicated that few TP proteins were expressed in colorectal carcinoma cells. The majority of cells that expressed TP activity were interstitial cells surrounding the cancer cells, especially the TAM. Consequently, few TP proteins were expressed in colorectal carcinoma cells, which were mostly TAM^9^, in the surrounding interstitial tissues. You *et al.*^10^ analyzed 28 specimens with colorectal carcinoma metastasis using immunohistochemical staining method and other methods. The results showed that the cells in colorectal carcinoma metastasis tissue that positively expressed TP were mostly TAM. Most results showed that TP activity was likely expressed by TAM. These findings have drawn the attention of several researchers, and similar studies are currently underway. Ren *et al.*^11^ conducted immunohistochemical staining analyses of 33 colorectal carcinoma specimens, and the results showed that TP was mostly expressed in cancer cells at the infiltrating edge. However, inflammatory cells (such as mononuclear cells, lymphocyte, and neutrophile granulocytes) in normal colorectal mucosa could also express TP. This result indirectly indicated that TP was mostly expressed by TAM in colorectal tissues. Kobayashi *et al.*^12^ observed the distribution of TP in cells using an electron microscope. The results showed that TP in colorectal carcinoma tissue was mainly distributed inside the particulate granules of macrophages. Makino *et al.*^13^ analyzed the genetic expressions of 43 colon cancer patients using laser capture microdissection and reverse transcription-polymerase chain reaction (RT–PCR) methods. The result showed that TP was expressed more in tumor stroma cells than in tumor cells.

In recent years, studies have indicated that TP activity was more likely to be expressed by TAM in colorectal cancer tissues. This finding has attracted considerable research attention. However, many factors affecting TP expression need to be further investigated.

## Relationship between TP and colorectal carcinoma treatment

TP has a vital function in colorectal carcinoma treatment: it catalyzes the conversion from 5-FU prodrugs (e.g., 5′-DFUR and CAP) to 5-FU that has antitumor activity. Three sequential enzyme reactions are present in this process. CAP is converted to 5′-deoxidation-5-flucytosine (dFCyd) under the action of carboxy-esterase in the liver after intestinal absorption, after which it is converted to 5′-DFUR by cytidine deaminase in the tumor tissue. When 5′-DFUR enters the tumor tissue, it is converted into 5-FU that has antitumor activity by TP. 5-FU functions in targeted-antitumor effect by blocking the DNA synthesis of tumor cells^3^. Slager *et al.*^14^ stated that TP is a target in oncotherapy processes with 5-FU and derivatives, and has an important function in growth, development, and metastasis processes of tumor. In 1998, Schwartz *et al.*^15^, transferred the INF gene into HT29 colorectal carcinoma cells, and found that the levels of TP proteins and mRNA increased. This laboratory finding indicated that INF could adjust TP expression during or after the transcription process. Thus, the conversion efficiency of 5′-DFUR to 5-FU increased, and the antitumor effects of 5′-DFUR were enhanced. In 2002, Nagata *et al.*^16^ analyzed the colorectal cancer cells with human TP transfection using MTT and other methods. The results showed that TP activity obviously increased compared with non-transfected cancer cells *in vitro* and *in vivo*. The sensitivity to 5′-DFUR increased, and the antitumor effects were enhanced. This finding is also indicated in Boskos’ study^17^.

Research findings have shown that colorectal carcinoma cells hardly express TP. Although 5-FU prodrugs are effective in colorectal carcinoma treatment, the transfer mechanism of drugs in cancer tissue remains unclear. Current studies support that the interstitial cells of cancer tissues, especially TAM, have an important function in conversion processes. Zhang *et al.*^18^ studied the antitumor effects of 5′-DFUR adjusted by two macrophage systems. The results showed that TP expressed by macrophage system transferred the 5′-DFUR to 5-FU, and consequently, 5-FU had an antineoplastic effect. This finding indirectly indicated that TP expression could strengthen the anticancer effect of 5′-DFUR. Zhang *et al.*^19^^,^^20^ analyzed the protein quantification and medium effective concentration on six colorectal carcinoma cell lines and two macrophage systems using ELISA, MTT, and other methods. The results showed that macrophages transferred 5′-DFUR to 5-FU; Thus, 5-FU could be released into the medium to produce a colorectal carcinoma cell growth-inhibiting effect. This result indicated that the macrophage could strengthen the antitumor effects of anticancer cells. Yasuno *et al.*^21^ analyzed 97 colorectal carcinoma specimens through immunohistochemical staining method. The findings indicated that patients with high TP expression and treated with fluorouracil exhibited better prognosis. This result indirectly showed that TP expressed in the mesenchyme could strengthen the antitumor effects of 5′-DFUR.

A number of chemotherapeutics, such as oxaliplatin, vorinostat, taxanes, and cyclophosphamide could improve TP expression in colorectal carcinoma cells. These drugs, together with fluorouracil, can produce a synergistic effect to improve the curative effect of chemotherapy. The study of Cassidy^22^ showed that CAP applied with oxaliplatin could improve TP expression and chemosensitivity with a synergistic effect. Gennaro *et al.*^23^ used RT-PCR, protein blot and immunohistochemical staining to prove that vorinostat with CAP could induce TP upregulation. This combined treatment also increased the collaborative anti-proliferation and cell apoptosis *in vitro*.

5-FU is a representative drug of colorectal carcinoma treatment that used to be the only therapeutic drug before 1985^24^. However, 5-Fu has significant toxic side effects, including diarrhea, nausea, vomiting, and myelosuppression. The development of biomedical technology resulted in the development of drugs with better therapeutic effects, such as CAP combined with oxaliplatin^22^. At present, 5-FU tends to be replaced by CAP as the best chemotherapeutic drug. On the other hand, CAP has some effects on patients who experienced 5-FU treatment failure. As an oral solution, CAP can simulate the antitumor effect of continuous intravenous drip of 5-FU. CAP is more convenient and simple to administer, prevents retention in vessels or chemotherapy pump, and has less side effects. Therefore, CAP is expected to be the drug of choice for the treatment of colorectal carcinoma^25^. Unger *et al.*^26^ showed that CAP and celecoxib could be used with radiotherapy to obtain a good therapeutic effect. Chiorean *et al.*^27^ indicated that the new adjuvant chemotherapy of combined utilization of CAP and irinotecan could also provide a good versus-tumor effect during the progressive stage of colorectal carcinoma treatment. Sadahiro *et al.*^28^ studied 76 patients with colorectal carcinoma surgical treatments to detect the responses of the patients to 5-FU/leucovorin (LV) or oral uracil and tegafur/LV. The results showed that a high expression of TP could increase body reactions to oral chemotherapeutics. Consequently, the curative effect of chemotherapy could be improved.

TP expression in colorectal carcinoma has a dual function. High expression of TP is related to poor prognosis factors of infiltration, growth, and tumor metastasis. Therefore, a number of studies concluded that TP inhibitors could help prevent angiogenesis and metastasis. 6-Amino-5-bromouracil is the classic TP inhibitor^29^. Studies in recent years indicated that 5-chloro-6-[(2-iminopyrrolidin-1-yl) methyl]uracil and 5-fluoro-6-[(2-aminoimidazol-1-yl)methyl]uracil are the two new developed and more effective TP inhibitors. The effect of the former is 1000 times better than that of 6-Amino-5-bromouracil^30^^,^^31^. Miyatani *et al.*^32^ stated that a combination of TP inhibitor and radiotherapy is effective for colon cancer. This result indicated that the TP inhibitor may function as a radiation sensitizer. In addition, TP is necessary for 5-FU prodrug activation. Promotion of TP expression in colorectal cancer tissue improves the curative effect of fluorouracil drugs, and this curative effect is important in colorectal carcinoma treatment. Therefore, increasing and decreasing TP expression in tissues have important effects on the emergence and development of tumors, as well as on the indices of treatment and prognosis.

## Summary

TP expression has dual influences on colorectal carcinoma. On one hand, excessive TP expression is related to infiltration, metastasis, and prognosis of colorectal carcinoma. Its promoting effect on angiogenesis based on enzymatic activity is also unique. On the other hand, TP is the key enzyme that contributes in the transformation from prodrug 5-FU to drugs with antitumor effects. TP also contributes in predicting toxicity of chemotherapy and sensitivity of colorectal cancer cells to 5-FU. TP is an effective target site which is the main focus for targeted therapy studies. However, further research is needed because many mechanisms of interactions between TP and other angiogenesis factors, as well as inhibiting factors, are still not clearly elucidated.
