# Erratum to research development of the relationship between thymidine phosphorylase expression and colorectal carcinoma

**DOI:** 10.7497/j.issn.2095-3941.2013.02.011

**Published:** 2013-06

**Authors:** Dian-Jun Ye, Ji-Min Zhang

**Affiliations:** Department of Gastrointestinal Surgery, The Second Affiliated Hospital of Guangzhou Medical University, Guangzhou 510260, China

In the published article^1^, an error appeared on page 10. The footnote about corresponding author should have read as follows:

Correspondence to: Ji-Min Zhang

Email: jimin0820@163.com

The authors and the journal apologize for the error and for any confusion it may have caused.
